# Polymer-Immobilized Photosensitizers for Continuous Eradication of Bacteria

**DOI:** 10.3390/ijms150914984

**Published:** 2014-08-25

**Authors:** Anton Valkov, Faina Nakonechny, Marina Nisnevitch

**Affiliations:** Department of Chemical Engineering, Biotechnology and Materials, Ariel University, Ariel 40700, Israel; E-Mails: anton_valkov@walla.com (A.V.); fainan@ariel.ac.il (F.N.)

**Keywords:** photodynamic antimicrobial chemotherapy, immobilization, photosensitizers, Rose Bengal, methylene blue, polystyrene, antibacterial activity

## Abstract

The photosensitizers Rose Bengal (RB) and methylene blue (MB), when immobilized in polystyrene, were found to exhibit high antibacterial activity in a continuous regime. The photosensitizers were immobilized by dissolution in chloroform, together with polystyrene, with further evaporation of the solvent, yielding thin polymeric films. Shallow reservoirs, bottom-covered with these films, were used for constructing continuous-flow photoreactors for the eradication of Gram-positive *Staphylococcus aureus*, Gram-negative *Escherichia coli* and wastewater bacteria under illumination with visible white light using a luminescent lamp at a 1.8 mW·cm^−2^ fluence rate. The bacterial concentration decreased by two to five orders of magnitude in separate reactors with either immobilized RB or MB, as well as in three reactors connected in series, which contained one of the photosensitizers. Bacterial eradication reached more than five orders of magnitude in two reactors connected in series, where the first reactor contained immobilized RB and the second contained immobilized MB.

## 1. Introduction

A deficiency in freshwater resources leads to serious water quality and quantity problems in the Middle East region. One way of solving, or at least reducing, this problem is to reuse wastewater for crop irrigation. However, pathogenic microorganisms in contaminated sewage and wastewater pose a threat to human health in the irrigated areas. The wastewater treatment includes a disinfection phase for eradication of pathogenic bacteria in order to prevent ecological and health hazards. The main existing methods of wastewater disinfection are based on the use of aggressive chemicals such as hypochlorites, chlorine dioxide and ozone, or power-consuming physical methods, such as UV radiation [1,2,3,4,5]. The disadvantages of these methods include the formation of mutagenic and carcinogenic byproducts in the disinfected water, mainly trihalomethanes and haloacetic acids [6,7,8,9], and high cost [10].

An alternative approach to the eradication of bacteria is based on photodynamic water treatment with the help of photosensitizers (PS), which are dye compounds capable of transferring the energy of absorbed visible light to surrounding bio-organic molecules (called a Type I photodynamic reaction) or to dissolved oxygen (called a Type II photodynamic reaction). The Type I reaction includes excitation of PS upon illumination at an appropriate wavelength and energy transition to organic substrates, thus, producing active free radicals and radical ions. The Type II mechanism differs from the Type I in the second stage of action, where energy is transferred from the PS to oxygen molecules, exciting them and causing the formation of reactive oxygen species. The active products in both cases cause direct and indirect damage to cellular components, such as membrane phospholipids and proteins, leading to membrane leakage, cytolysis, and death of pathogenic cells. PS were found to be inactive and harmless to cells when applied in the dark [11,12].

There are several advantages to using PS for wastewater disinfection: PS molecules are harmless to human beings and animals [11], the disinfection process does not require an energetic impact since sunlight can be used for illumination. Furthermore, PS do not only inactivate bacteria, but also sewage bacteriophages [13], and promote photolysis of trace organic contaminants in the wastewater [14,15,16,17]. Resistance of bacteria to PS has not been described to date, thus, increasing the feasibility of using photodynamic treatment on hospital wastewaters contaminated with multi-drug resistant bacterial strains [18].

The suitability of free PS for wastewater disinfection was shown by Jemly *et al*. [19], Carvalho *et al*. [20], Jori *et al*. [21], Sabbahi *et al*. [10], and Almeida *et al*. [18]. Although PS in solutions demonstrated high efficiency in killing fecal coliforms and streptococci, it should be noted that use of PS in solutions is problematic, due to the need for constant addition of PS to the treated wastewater and the necessity of removing the PS after the treatment. These problems can be solved by immobilization of the PS onto a solid phase. Attempts to apply immobilized PS for wastewater disinfection and for production of antibacterial surfaces were described by Jiménez-Hernández *et al*. [22], Bonnett *et al*. [23], Villén *et al*. [24], Manjón *et al*. [25,26], Sabbahi *et al*. [10], and Cahan *et al*. [27]. For this purpose, PS were attached onto solid supports, such as chitosan, polyester isophtalic resin, porous poly(dimethylsiloxane) (silicone), cationic nylon, polyethylene, poly(vinylidene difluoride), and cellulose membranes by adsorption, dissolution and casting, covalent bonding or by an electrostatic interaction. The list of immobilized PS includes methylene blue (MB), toluidine blue O, Rose Bengal (RB), 5,10,15,20-tetrakis (*p*-hydroxyphenyl)porphyrin, 5,10,15,20-tetrakis (*p*-aminophenyl)porphyrin, zinc(II) phthalocyanine tetrasulfonic acid, tris(4,4'-diphenyl-2,2'-bipyridine)ruthenium(II), tris(4,7-diphenyl-1,10-phenanthroline)ruthenium(II), tris(1,10-phenanthrolinyl-4,7-bis(benzenesulfonate)ruthenate(II) and tris(4,40-dinonyl-1,10-phenanthroline)ruthenium(II).

All immobilized PS exhibited good photokilling abilities on a model of Gram-negative and Gram-positive bacteria (*Escherichia coli* and *Enterococcus faecalis*, respectively) and wastewater fecal bacteria in a batch and in a continuous regime or with repeated use. Immobilized PS demonstrated higher stability and resistance against photobleaching than free PS, and maintained at least some of their antibacterial activity after storage for several months in the dark [23,26]. In most cases, the immobilized PS did not leak from the solid supports. However, MB immobilized at a high loading exhibited fast release from the polymer [10]. The lifetimes of reactive oxygen species were shown to be ten times longer when photogenerated by immobilized PS than by PS dissolved in water [26]. It was proven that eradication of bacteria by immobilized PS was performed with singlet molecular oxygen [22,26] or by a combination of Type I and Type II mechanisms [10].

In our previous works we described a series of batch experiments in which the photodynamic antibacterial activity of RB and MB was studied when these photosensitizers were in a free form, encapsulated into liposomes or immobilized in polymers [28,29,30,31,32,33,34,35,36,37]. In the present work we report the continuous photoeradication of Gram-positive *Staphylococcus aureus*, Gram-negative *E. coli* and wastewater coliform bacteria by MB and RB immobilized in polystyrene.

## 2. Results and Discussion

Two immobilized PS, RB and MB, were used for the experiments on continuous eradication of bacteria. A rapid, simple and effective method was chosen for immobilization. This method was based on the dissolution of a PS and a polymer in the same solvent, application of the solution onto surfaces and evaporation of the solvent. This procedure led to the inclusion of more than 99% of the PS into thin (15–140 µm) porous polymeric films [34,37]. The surfaces with the immobilized PS were thoroughly washed in the dark under control of absorbance of washings at the appropriate wavelength as described previously in order to exclude a possible effect of PS leakage from the polymer [34]. Properties and characteristics of the immobilized in polystyrene photosensitizers were examined and described in our previous work [34].

Earlier we have shown principal suitability of PS immobilized by this method for inactivation of Gram-positive and Gram-negative bacteria [34,37], by comparing three types of polymers—polystyrene, polycarbonate and poly(methyl methacrylate). PS immobilized in polystyrene were found to exhibit the best antibacterial properties [37]. Polystyrene was, therefore, chosen in the current work as the polymeric support for immobilization.

**Figure 1:**
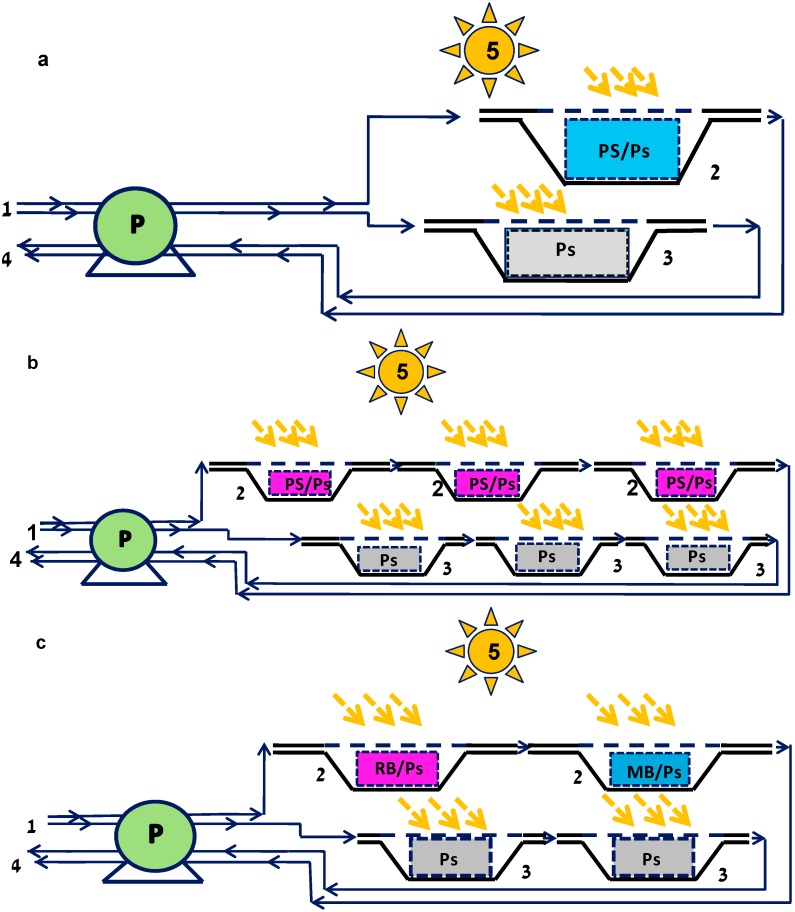
The scheme of the photoreactors for continuous eradication of bacteria.(**a**) Single-reservoir reactor, bottom-coated with MB or RB immobilized in polystyrene (PS/Ps); (**b**) Triple-reservoir reactor, where each reservoir was bottom-coated with MB or RB immobilized in polystyrene (PS/Ps); (**c**) Double-reservoir reactor, where the first reservoir was coated with RB immobilized in polystyrene (RB/Ps) and the second was coated with immobilized MB (MB/Ps). 1—Inlet; 2—Experimental reservoirs; 3—Control reservoirs coated with polystyrene (Ps); 4—Outlets; 5—A white light source. P—A multichannel peristaltic pump.

A photoreactor for continuous inhibition of suspended bacteria was designed based on one to three shallow reservoirs which were bottom-coated with immobilized PS and control reservoirs that were coated with polystyrene without PS (Figure 1). All reservoirs were illuminated from above with a white lamp emitting visible light in the range of 400–700 nm, which was proven by registration of the emission spectrum [34].

In the first stage of the research we studied the possibility of inactivating model bacteria—Gram-positive *S. aureus* and Gram-negative *E. coli*—in a continuous regime. Suspensions of the bacteria were passed in parallel through two shallow reservoirs—one with a polystyrene coating of immobilized MB or RB, and a control reservoir (Figure 1a).

**Figure 2:**
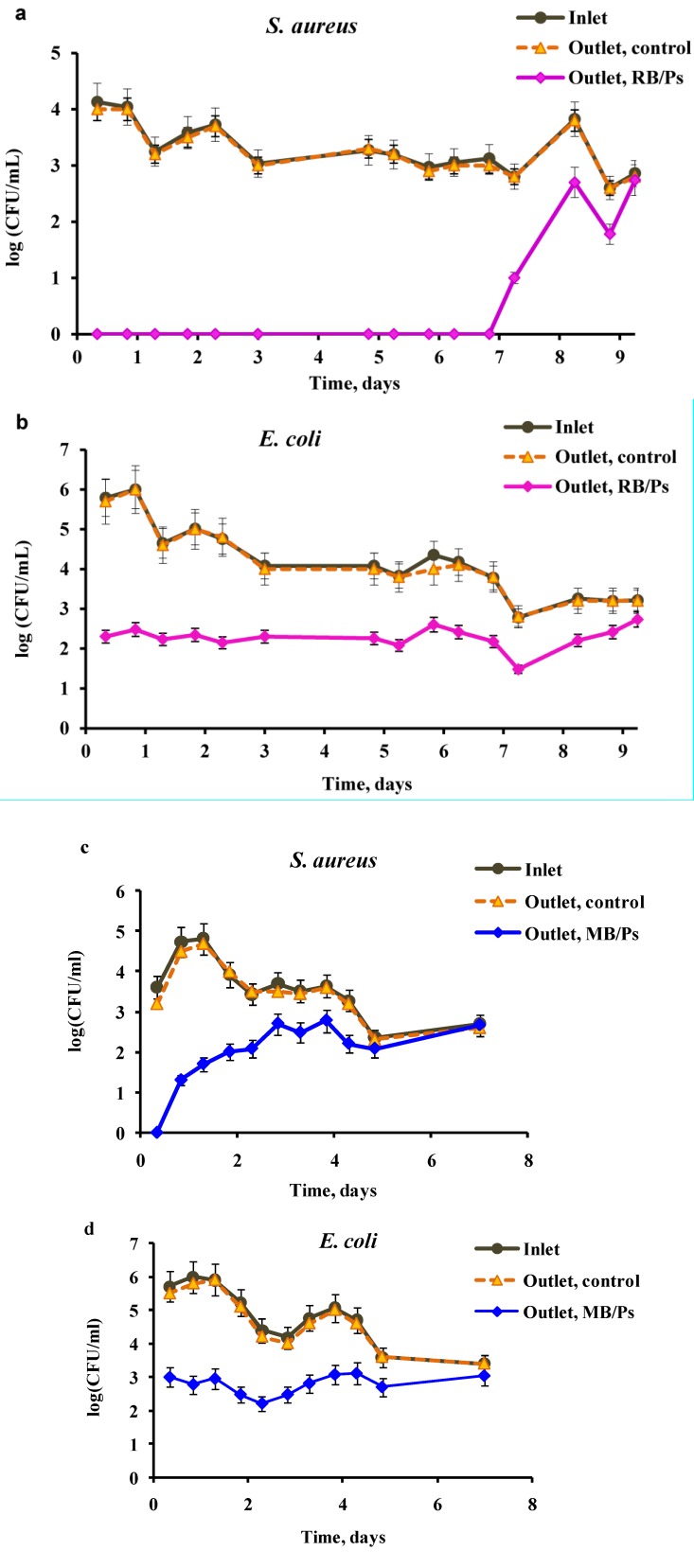
Effect of PACT on viability of *Staphylococcus aureus* (*S. aureus*) (**a**,**c**) and *Escherichia coli* (*E. coli*) (**b**,**d**). Suspensions of bacterial cells passed through a photoreactor equipped with a 200 mL reservoir bottom-coated with RB immobilized in polystyrene (RB/Ps, (**a**,**b**)) or MB (MB/Ps, (**c**,**d**)) at a rate of 1 mL·min^−1^under illumination with 1.8 mW·cm^−2^ white light. The scheme of the reactor is presented in Figure 1a. The suspensions were sampled at the inlet of the reactor and at the outlets from the experimental reservoirs (Outlet, RB/Ps or Outlet, MB/Ps) and from the control reservoir (Outlet, control).

The results of this experiment are presented in Figure 2. The bacterial concentration was not affected at the outlet of any of the control reservoirs. In contradistinction, the concentration of cells at the outlets of the experimental reservoirs decreased by several orders of magnitude. Immobilized RB totally inhibited *S. aureus* and caused a 2 to 3 log_10_ drop in the *E. coli* concentration for a period of one week (Figure 2a,b). The effect was less prolonged in the reservoir with coatings of immobilized MB: The concentration of both *S. aureus* and *E. coli* decreased by 1 to 3 log_10_ for only four days. A possible reason for the limited time during which the immobilized PS was active is the phenomenon of PS photobleaching, which is well-known for free PS species [10,19].

**Figure 3:**
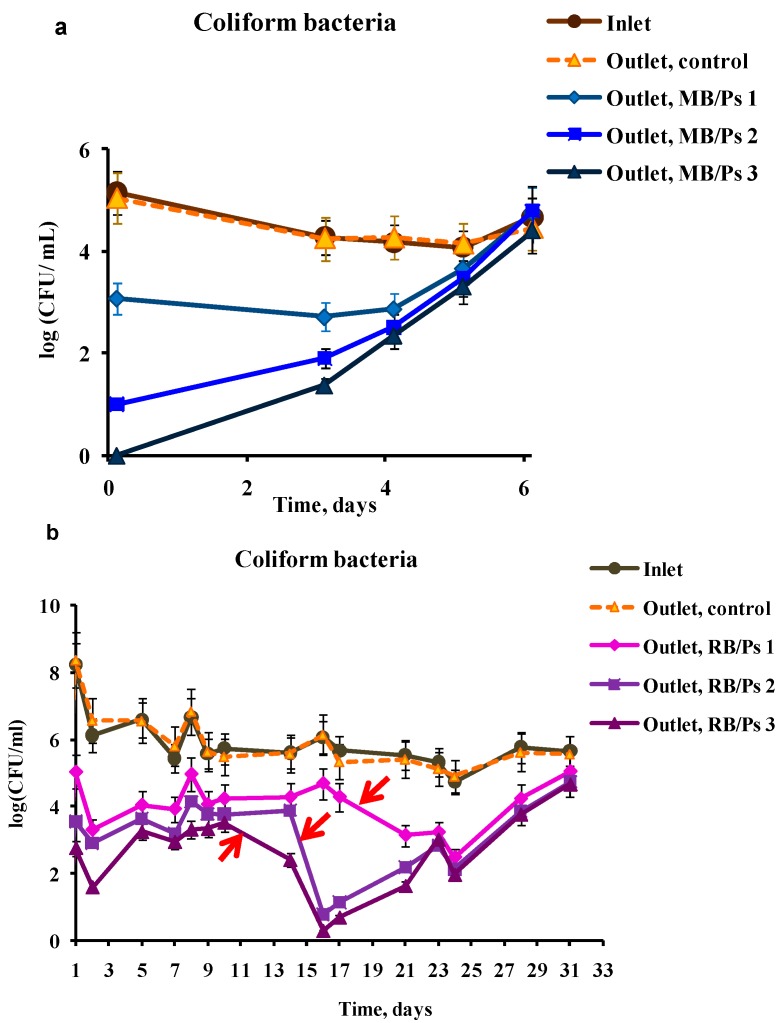
Effect of photodynamic treatment on the viability of coliform wastewater bacteria. Wastewater after primary treatment was passed through a photoreactor consisting of three 200 mL reservoirs that were bottom-coated with MB (MB/Ps) immobilized in polystyrene (**a**) or RB (RB/Ps); (**b**) connected in series at a rate of 1 mL·min^−1^ under illumination with 1.8 mW·cm^−2^ white light. The scheme of the reactor is presented in Figure 1b. The suspensions were sampled at the inlet of the reactor and at the outlets from each experimental reservoir ((**a**)—Outlet, MB/Ps 1, MB/Ps 2 and MB/Ps 3; and (**b**)—Outlet, RB/Ps 1, RB/Ps 2, and RB/Ps 3) and from the control reservoir (Outlet, control). For the RB/Ps, the polymer coatings were replaced on day 11 in the third reservoir, on day 15 in the second reservoir and on day 18 in the first reservoir (shown by red arrows in graph (**b**)).

In an attempt to improve the efficiency of the photoreactor, we constructed another scheme based on three reservoirs that were bottom-coated with immobilized PS and were connected in series (Figure 1b). As in the previous experiment, the control reservoir was coated with polystyrene without addition of PS. The retention time of bacterial suspensions in the control reservoir was equal to that in the experimental reservoirs. In this experiment, the viability of wastewater coliform bacteria was examined and the bacterial concentration was tested at the inlet to the reactor and at the outlet of each of the three reservoirs. Figure 3 shows the results of this experiment. Immobilized MB (Figure 3a), as well as immobilized RB (Figure 3b), showed high ability to eradicate coliform bacteria, whereas bacteria that passed through the control reservoir remained unaffected. Each of the subsequent experimental reservoirs impacted the overall efficiency of the photoreactor and the bacterial concentration decreased from the first to the third reservoir outlets. As in the experiment with the single-reservoir photoreactor (Figure 2c,d), immobilized MB showed a rather short-term efficiency, and actually ceased functioning after four to five days. We attribute the observed decrease in activity to photobleaching of PS rather than to their leakage from the polymeric support. According to our previous data [34], after first several washings performed before the usage of the PS-containing films, leakage of PS from the support did not occur or at least, could be considered negligible. Taking into account the reduction in activity of immobilized PS in time, the further experiments were planned with an option of replacing the reservoirs with immobilized PS, which ceased to affect the bacterial concentration. The experiment was run for the first ten days without replacing RB-polystyrene coatings and the decrease in the bacterial concentration ranged between five orders of magnitude in the first two days to two orders on day 10. However, on days 11, 15, and 18 the coatings were replaced with fresh ones in the third, second and first reservoirs, respectively, and the eradicating ability of the entire system was recovered each time, as can be seen by sharp drops in the coliform concentration. These manipulations enabled prolongation of the effective operation of the entire system for up to 24 days (Figure 3b), contrary to a one-week period observed in the case of the single-reservoir reactor (Figure 2a,b). Regular replacement of the PS-polystyrene coatings in the photoreactor is a good solution for the problem of PS photobleaching and opens new vistas for unlimited continuous disinfection of wastewater.

**Figure 4:**
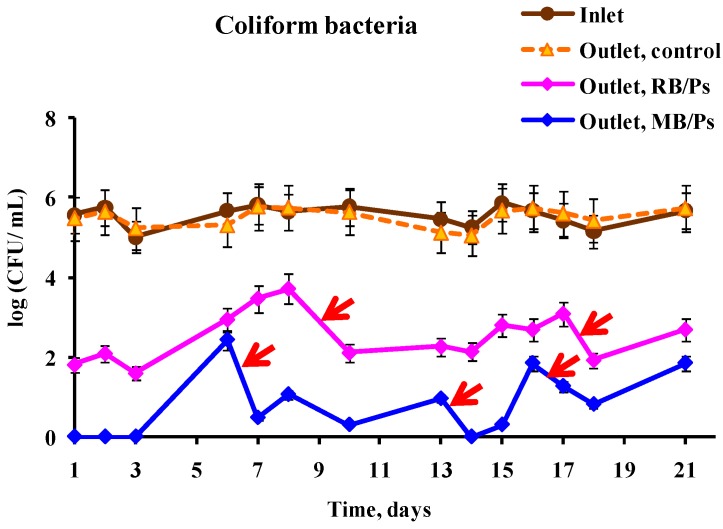
Effect of photodynamic treatment on the viability of coliform wastewater bacteria. Wastewater after primary treatment was passed through a photoreactor consisting of two 200 mL reservoirs that were bottom-coated with immobilized in polystyrene RB (RB/Ps, the first reservoir) and MB (MB/Ps, the second reservoir) connected in series at a rate of 1 mL·min^−1^ under illumination with 1.8 mW·cm^−2^ white light. The scheme of the reactor is presented in Figure 1c. The suspensions were sampled at the inlet of the reactor and at the outlet of each experimental reservoir (Outlet, RB/Ps and Outlet, MB/Ps) and from the control reservoir (Outlet, control). In the first reservoir, the RB/Ps polymer coatings were replaced on days 9 and 17; and in the second reservoir the MB/Ps coatings were replaced on days 6, 13, and 16 (shown by red arrows on the graph).

Another approach to increasing the efficiency of coliform bacterial eradication was based on a combination of reservoirs with coatings that contain different PS. For this purpose, a two-reservoir photoreactor was constructed. The first reservoir was coated with immobilized RB and the second with immobilized MB (Figure 1c). This experiment also included replacement of coatings with fresh ones in order to prolong the photoreactor’s activity. Figure 4 presents the results of coliform inhibition by this system. As in the previous experiments, viability of the bacteria was not decreased in the control reservoirs. However, the situation in the experimental system was quite different: the bacterial concentration at the outlet from the first (RB-containing) reservoir decreased by 2 to 4 log_10_, and at the outlet from the second (MB-containing) reservoir it dropped by an additional 1–3 log_10_, so that the average coliform outlet concentration during the three-week period of the continuous experiment was 640 ± 160 CFU/100 mL. Taking only samplings of every fourth day following the replacement of the MB-containing coating into account, the total coliform concentration did not exceed 260 ± 140 CFU/100 mL. In any case, effluents from the photobioreactor satisfied the regulations of the World Health Organization (WHO) which limit fecal coliform bacteria in unrestricted irrigation to 1000 fecal coliform bacteria/100 mL, and in restricted irrigation to 10^5^ fecal coliform bacteria/100 mL [38]. It should be noted that the WHO regulations refer only to fecal coliforms, which are only a part of the total coliform population. It can be concluded that the best results in continuous eradication of coliform bacteria were obtained by a combined treatment with two different immobilized PS, provided that the RB-polystyrene coating is replaced once a week and the MB-polystyrene coating every four days.

The possibility of using free and immobilized PS for disinfection of wastewater has been studied by several groups. Free MB, RB, (4-*N*-methyl-pyridyl)porphyrin tetra-tosylate and several other cationic derivatives of porphyrin were shown to be effective against fecal coliforms under sunlight illumination or white lamplight (PAR radiation) [19,20]. The authors registered a 1 to 4 log_10_ decrease in the coliform concentration. Sabbahi *et al*. [10] compared the inhibition rates of several fecal species (coliform, *E. coli*, streptococci) treated with free MB under sunlight and white artificial illumination and found no difference between the illumination methods. However, the inhibitory effect of MB was strongly dose- and pH-dependent in all cases. The authors also immobilized MB in a polyester-isophtalic resin and performed experiments on batch photoinactivation of fecal coliform and streptococci species, replacing the wastewater every 3 h. A maximal 2.5 log_10_ decrease in bacterial concentration was achieved in all experiments, which was observed during a 3–27 h period [10]. It must be noted that, in the present work, we report much higher stability and lifetime of the immobilized MB, which maintained its antibacterial activity for up to four days. Immobilized RB exhibited even higher stability and was found to be active for up to seven to eight days, with up to 99.99% eradication of *S. aureus* and up to 99.9% eradication of *E. coli* cells under moderate illumination for 3 h. These data can be compared to the results obtained using PS based on derivatives of Ru(II). The latter were shown to inactivate 99% of *E. coli* and 99.9% of *E. faecalis* in a solar photocatalytic reactor, which contained strips of the immobilized PS, after 5 h of continuous circulation of bacterial suspensions at initial concentrations of 10^2^ to 10^4^ CFU·mL^−1^ [24]. Bonnett *et al*. [23] tested a photoreactor based on Zn-containing PS immobilized on chitosan membranes for water disinfection. When the initial concentration of cell suspensions was 10^5^ cells·mL^−1^, 99.6% of *E. coli* cells were destroyed after almost 3 h of circulation under illumination by a halogen lamp. It should be mentioned that the system proposed by us was at least as efficient for bacterial eradication.

Jiménez-Hernández *et al*. proposed six characteristics of supports suitable for immobilization of PS: Compatibility with the PS; good rheological features; mechanical strength and stability towards light; good oxygen permeability; high biocompatibility; commercial availability [22]. The described polystyrene support meets these demands. Polystyrene is totally compatible with the PS tested in this work, since the structures of these species include aromatic rings. The obtained polymeric films have a porous structure confirmed by electron microscopic investigations [34,37], and such a structure should exhibit high oxygen permeability. Biocompatibility of polystyrene and its ability for good adhesion of bacterial cells was shown in our earlier work, where it was demonstrated that bacterial cells grow well on the surface of polystyrene and even develop biofilms [34]. Regarding commercial availability, polystyrene is considered to be a common, available and low-cost material. Rheological characteristics, mechanical strength and stability of polystyrene towards visible light are high enough. However, they are irrelevant in the proposed reactor’s configurations, since the polystyrene films are used for coating the bottom of reservoirs through which wastewater flows at a very low rate and the period of usage is limited to four to eight days.

## 3. Experimental Section

### 3.1. Immobilization of Photosensitizers (PS) in Polystyrene

Immobilization of PS was performed as described in [34]. Briefly, 25 g polystyrene were dissolved in 100 mL of chloroform and mixed with a solution of 250 mg PS (methylene blue (MB) or Rose Bengal (RB)) in 20 mL chloroform. The solutions were dispensed into disposable polycarbonate (15 × 10 × 2 cm) reservoirs in 12 mL portions and the solvent was air-evaporated in a dark hood, producing a thin layer of polymer. After drying, the polymer film was washed with distilled water to remove free PS. The washings were collected, the non-included PS portion was determined by measuring absorption at the appropriate wavelength for each PS, and the degree of PS inclusion into the polymer was determined [34]. The coated reservoirs were stored in the dark until use.

### 3.2. Bacterial Growth

Cultures of *S. aureus* (ATCC 25923) and *E. coli* (ATCC 10798) were grown on brain-heart agar (BH, Acumedia, Baltimore, MD, USA) for 24 h, transferred into brain-heart infusion broth (BH, Acumedia, Lansing, MI, USA) and grown again at 37 °C and shaking at 170 rpm. Cells were harvested by centrifugation, washed twice with 0.05 M phosphate-buffered saline (PBS), pH 6.5, diluted with PBS up to OD_660_ = 0.1 and then serially diluted in two to four decimal dilutions.

### 3.3. Wastewater Sampling

Wastewater samples were taken from the municipal wastewater collector of the Ariel University (Ariel, Israel) after primary treatment and were used for experiments on the day of sampling.

### 3.4. Continuous Photoreactor for Wastewater Treatment by Polymer-Immobilized PS

Bacterial suspensions or wastewater samples at concentrations of 10^4^ or 10^8^ cells·mL^−1^ in sterile saline were passed through a photobioreactor containing one to three 200 mL reservoirs that were bottom-coated with the PS-polymer connected in series, under illumination with a white luminescent lamp at the intensity of 1.8 mW·cm^−2^. Control experiments were carried out under the same conditions, but in a reactor in which the reservoir was bottom-coated with polystyrene only. The bacterial suspensions were fed and discharged at a flow rate of 1 mL·min^−1^ supplied by a multichannel Ecoline peristaltic pump (Ismatec, Glattbrugg, Switzerland). For evaluation of bacterial concentrations of *S. aureus* or *E. coli*, samples at the inlets and the outlets were diluted at various decimal dilutions with saline and 100 µL aliquots were spread over BH-agar Petri dishes, which were incubated at 37 °C overnight, after which CFU (colony forming units) were counted. For the wastewater experiments, the total coliform concentration was determined as follows: 11 mL samples of wastewater before and after the treatment were diluted with distilled water at various decimal dilutions. 100 mL of the sample were transferred through a nitrocellulose membrane filter with a pore diameter of 0.45 µm (Millipore, Tullagreen, Carrigtwohill, Ireland). Filters were placed, top side up, on the surface of the m-Endo agar (Fluka, Ahmedabad, Gujarat, India) in Petri dishes and incubated at 37 °C overnight. Red-colored colonies having metallic shine were counted, and the concentration of total coliform bacteria was determined.

### 3.5. Statistical Methods

The results obtained from at least three independent experiments carried out in duplicates were statistically analyzed by ANOVA single-factor or ANOVA two-factor analyses. The difference between the results was considered significant if the *p*-value was less than 0.05.

## 4. Conclusion

Rose Bengal and methylene blue immobilized in polystyrene showed high efficiency in continuous inactivation of Gram-positive *S. aureus*, Gram-negative *E. coli* and wastewater coliform bacteria.

Combining two continuous photoreactors containing immobilized Rose Bengal and methylene blue in series enabled the achievement of deep (more than five orders of magnitude) eradication of wastewater coliform bacteria.
